# Infection Spread and Outbreaks Support with Spatial-Temporal Visualization Tool for Hospitals

**DOI:** 10.1007/s10916-025-02219-7

**Published:** 2025-06-18

**Authors:** Denisse Kim, Bernardo Canovas-Segura, Manuel Campos, Sergio Aleman Belando, Jose M. Juarez

**Affiliations:** 1 Med AI Lab, University of Murcia, Campus Espinardo, 30100 Murcia, Spain; 2 Murcian Bio-Health Institute (IMIB-Arrixaca), El Palmar, 30120 Murcia, Spain; 3 J. M. Morales Meseguer General University Hospital, Murcia, 30008 Murcia, Spain; 4 Ciber Fragilidad y Envejecimiento Saludable (CIBERFES), Av. Monforte de Lemos, 28029 Madrid, Spain

**Keywords:** Visualization application, Infection control, Spatial-temporal visualization, Multi-resistant bacteria, Hospital-acquired infection

## Abstract

Hospital-acquired infections (HAIs), especially those caused by multidrug-resistant bacteria, represent a critical challenge, increasing healthcare costs, hospital stays, and mortality rates. Monitoring HAIs requires integrating spatial-temporal data from patient records and microbiology results. However, current manual methods are time-consuming and error-prone. Although temporal factors are often considered, spatial patient distribution and hospital topological factors are frequently overlooked. Interactive information visualization provides a solution, combining diverse data sources to enhance understanding of spatial-temporal relationships. We aim to develop *OBViz*, an interactive visual tool employing spatial-temporal visualization techniques to analyze infection spread and hospital epidemic situations over time. Four user tasks relevant in HAIs control were defined, focusing on spatial-temporal pathogen localization and identifying outbreak origins. Using Unity 3D and C#, along with a simulated dataset of hospitalized patients experiencing an infection spread, we developed a visual interactive tool that integrates 3D hospital visualization for patients’ individual monitoring, 2D visualization for tracking epidemiological indicators, and tabular view for detailed information. A user study with 14 healthcare personnel evaluated its usability, usefulness and interpretability. Interactivity and animations accurately depicted movements and infection processes, while known charts facilitated temporal understanding. Despite room for improvement in patient tracking (57.14% success rate), OBViz demonstrated strong potential for decision-making (91.43% success rate), healthcare education, and integration into clinical workflows (*Post-Study System Usability Questionnaire* result: 1.85). The tool’s interactive spatial visualization and clear time control were preferred over more abstract methods, highlighting its utility in hospital epidemic analysis.

**Supplementary Information:**

The online version contains supplementary material available at 10.1007/s10916-025-02219-7.

## Introduction

Multidrug-resistant (MDR) microorganisms are those that have evolved and developed resistance to drugs used to treat them [1]. This makes their treatment complex and increases hospital stays, costs, mortality, and morbidity [2, 3]. Hospital-acquired infections (HAIs) caused by these microorganisms are especially dangerous and a growing priority for healthcare systems [4].

Controlling and preventing these infections requires monitoring in healthcare settings, where clinicians manually correlate patients’ spatial location at any time with microbiological test results. The large amount of clinical data generated daily for each patient makes this process complex, error-prone and time consuming for specialists and hospital administrators [5].

Applying interactive Information Visualization techniques in decision-support systems can mitigate this information overload [6] and improve the discovery of trends, patterns and patient clusters. However, epidemiological visualization tools typically focus on population-level disease surveillance over geographic areas [7, 8]. Few advances analyze individual-level infection spread within building interiors over time [9]. These tools would benefit epidemiologists, administrators and less experienced clinicians, and help in training for complex epidemic situations like the COVID-19 pandemic.

**Table 1:** Decomposition and abstraction of user tasks

Task	High Level	Medium Level	Low Level
T1: Analysis of the epidemiological situation in various places in the hospital over time using indicators	Discover information: generate or verify hypotheses	Explore trends, characteristics in the data	Compare places
T2: Detection of sources of infection over a period of time	Discover information: discover concentrations of cases	Locate the location of concentrations	Identify concentrations in space
T3: Detection of patient zero	Discover information: generate or verify hypotheses	Locate index case: known target, unknown location	Identify index case
T4: Study of possible outbreaks	Discover information: discover concentrations of cases	Explore concentrations in time and space	Summarize concentrations

In contact tracing and epidemiological studies, [10] designed multiple specialized views and a storyline visualization to analyze transmission pathways, patient contacts, and outbreak progression. Müller et al. [11] applied a recurrent neural network model to detect possible infections and transmissions, proposing a visual interface to explore the results. Sondag et al. [12] visualized time-varying infection maps with representative trees and interactive techniques for evaluating control policies.

Regarding spatial topology and space use, these approaches often involve sensors, video cameras and mobile phones. Oppermann et al. [9] introduced visual decision support tools for occupancy data in building management, visualizing trajectory-free spatial-temporal data in indoor environments. Boudreault et al. [13] proposed a situation-awareness dashboard displaying Intensive Care Unit (ICU) beds and information on staff and equipment to improve resource management and decision-making.

Our goal is to design spatial-temporal visualization techniques that facilitate the exploration of infection spreads within hospitals. For this reason, we develop an interactive visual tool, *OBViz* (OutBreak Visualization), to help analyze the epidemic situation of a hospital over time, allowing an understanding of the information contained in epidemiological indicators focusing on spatial and temporal dimensions.

## Methods

### Input Data

Monitoring an infection and its spread requires chronological information about patients’ spatial locations and microbiological test results. However, collecting these data for each individual presents challenges (eg. data quality, bias, confidentiality, and privacy protection [14]). Therefore, real publicly accessible datasets are not feasible for this purpose.

To address this, we used a highly realistic artificial dataset of hospitalized patients, generated by a simulator we developed and validated clinically [15]. It represents the spread of a *Clostridioides difficile* infection (CDI) in a hospital. CDI is a leading cause of infectious diarrhea in hospitalized patients, with increasing rates of resistance, mortality and hospitalizations [16]. Its incidence reached 92 per 100,000 inhabitants in North America and Europe [17], and its treatment is increasingly expensive and continues to be debated.

The synthetic data^1^ includes individual-level patient information from admission to discharge or death from CDI. Every 8 hours, it records the patient’s health state and bed location, enabling room-sharing tracing. Health states are based on an adaptation of the SEIRD (Susceptible, Exposed, Infected, Recovered, Deceased) model [18]. We added colonized upon admission and the Non-Susceptible status (“SEIRD-NS” model), representing patients with immunity to the disease. More information on the simulator and data in Appendix A.

### User Tasks

Following Centers for Disease Control and Prevention recommendations for preventing and controlling MDR infections in healthcare settings [19], we compiled daily hospital tasks related to HAI control, focusing on spatial-temporal pathogen localization and outbreak identification. Using Munzner’s methodology [20], we abstracted these tasks to model clinical experts’ actions as visualization tasks. Each action was decomposed into three levels of detail (see Table 1).

**Fig. 1:**
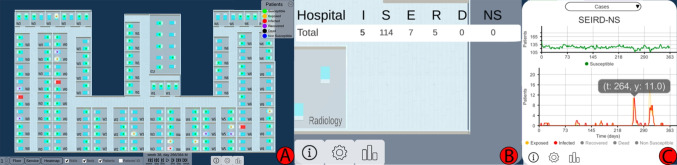
The 3 views of OBViz. (A) The Hospital view shows the patients and the spread of the disease in a 3D spatial-temporal representation. (B) The Tabular view shows the values of the epidemiological indicators by place. (C) The Epi view displays the temporal evolution of the epidemiological indicators

### Tools and Architecture

We developed the tool using Unity 3D (v2021.3.15f1) and C#, featuring 3D hospital visualization. Data visualization employed the E2Chart asset (v3.2) from Ice Pond Studio [21]. Through interactions with the environment and cameras, we enable both a general analysis of the hospital from different perspectives and a detailed analysis of patient interactions at a low level of abstraction, facilitating disease monitoring. We used Blender (v3.6) to model hospital elements and PostgreSQL (version 15.4) for data persistence.

### User Experiment Design

To ensure the OBViz tool met users’ needs, we conducted a user-centered study with healthcare personnel to assess its usability, usefulness and interpretability. To design and validate this study, infectious disease expert performed a preliminary test to correct errors from a clinical perspective. Moreover, three collaborators with medical informatics background tested it to ensure the evaluation content was clear and to identify any necessary wording or organizational changes. The resulting study, conducted in the meeting room of the Infectology Department of the J. M. Morales Meseguer Hospital, consisted of three stages:

#### 1. Introduction

Participants were introduced to the visual model, tool’s objectives, SEIRD-NS model, epidemiological indicators and the scenario they would work with. This scenario consists of a simulation of a year with several CD outbreaks. We conducted a tutorial on the tool to demonstrate the functionalities with a scenario different from the one they would use later. The entire explanation lasted approximately 15 minutes (transcription in Appendix B).

#### 2. User experiment

Participants interacted individually with the tool and answered questions about the scenario while thinking out loud about what they were doing. This allowed us to understand what actions they wanted to perform at each moment, what they felt was missing, or what they would like to see instead. Each participant performed the evaluation individually using a Lenovo Legion Slim 7 and a 27” 75 Hz IPS AOC monitor. First, we let them familiarize themselves with the tool for several minutes and then the experiment began. Data such as age, gender, specialty, and experience were collected. Participants could stop at any time and ask questions, without a time limit imposed. This stage allowed us to evaluate the interpretability of the tool (questions regarding the CDI scenario in Appendix C).

#### 3. User feedback

Participants completed a satisfaction questionnaire and provided suggestions for future developments. The questionnaire was based on the *Post-Study System Usability Questionnaire* (PSSUQ) v2 standard [22] and consisted of 18 questions: the first 8 referring to the usability of the system, 9 to 14 referring to the quality of the information presented, and 15 to 18 referring to the quality of the interface. The Likert scale used in this questionnaire had 7 levels, from Completely agree (1) to Completely disagree (7), or Don’t Know (8) (see Appendix D). Suggestions and feedback were collected during informal conversations with each participant after completing the evaluation [23].

## Results

### Tool Design

In this section, we present the interactive visual tool, which consists of three strategies to display spatial-temporal information (called *views*): Hospital, Tabular and Epi view (Fig. 1). We explain their implementation and the interactions between them and with the user. See Supplementary File 1 for an example of the interactions and functionalities of the tool.

#### Hospital View

The Hospital view shows pathogen spread, intra-hospital patient transfers, and endemic evolution across services on a 3D hospital plan (Tasks 1-3). This enables analysis of patients’ spatial distribution at each time, infection locations, contact events (when and with whom they shared a room) and areas with high patient concentrations over time.

**Fig. 2:**
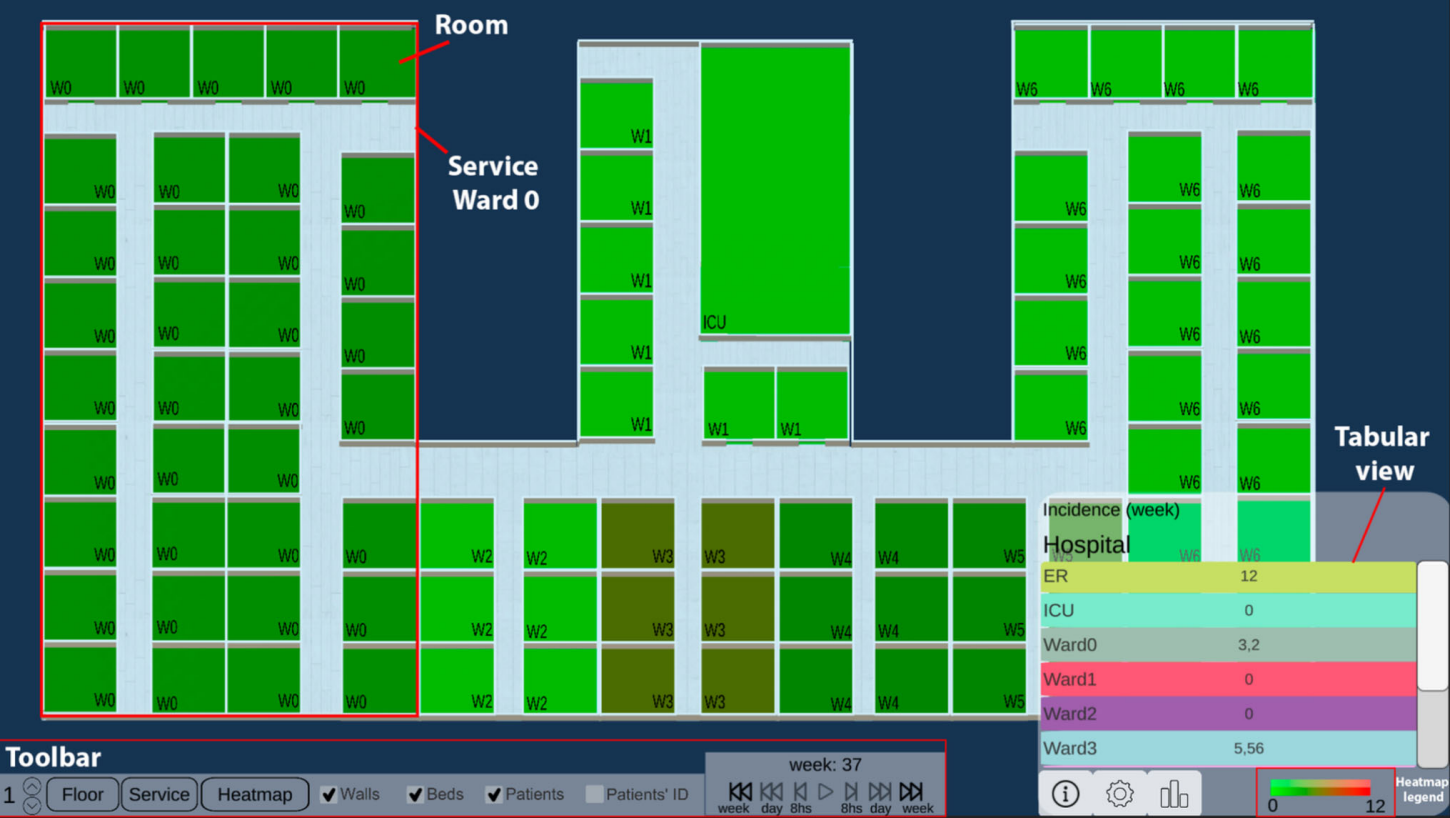
Heatmap function. It aggregates the epidemiological indicator by week and service, and shows the values in the tabular view

Patients’ health states, based on the SEIRD-NS model, represent the health transitions regarding a CDI. For quick identification, they are coded with a segmented color scale, following Aigner et al. [24]: green (susceptible), yellow (exposed), red (infected), purple (recovered), black (deceased), and blue (non-susceptible). This helps identify transmissions (ie. when a susceptible patient was in contact with an infected one and became exposed), and potential future cases that could increase the spread.

Users can minimize other views to focus on the hospital and interact with the camera to move, zoom and rotate. To avoid visualization issues like occlusion and perspective distortion common in 3D representations [25], we implemented an orthographic camera, rendering objects uniformly without perspective.

Through a toolbar, users can control patient movements and infection progression animations (forward, backward, pause and resume). They can track patient locations, infection evolution, patients duration in each place, and contacts. Users can also skip time intervals (every 8 hours, day or week), instantly showing patients’ new locations at that moment. Time information (week, day, and morning/afternoon/night) is displayed above these controls. Users can switch floors of the hospital, hide non-essential visual objects (beds or walls), activate patient IDs to identify them, and group the Tabular view information by floor or service. They can enable the HeatMap functionality to see weekly-aggregated epidemiological indicators and track the evolution of each service (Fig. 2). This is useful for viewing in a controlled manner, summarized information on the status of each service (eg., to easily identify services where a given epidemiological threshold (eg. prevalence) is surpassed, locate extreme values, or compare the incidence rate between different wards).

#### Tabular View

**Fig. 3:**
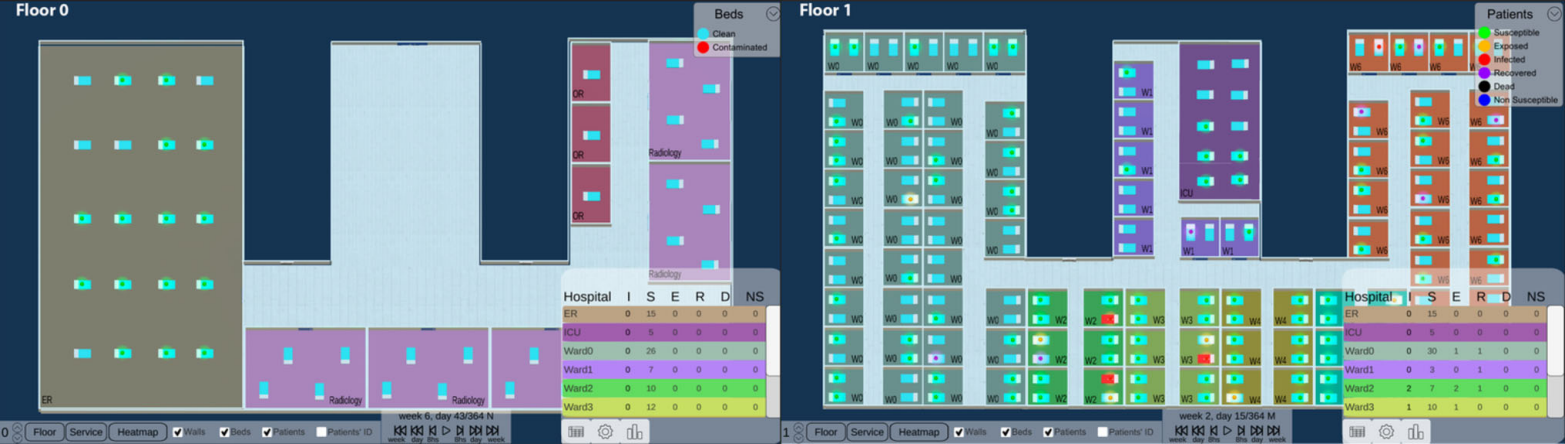
Epidemiological indicator aggregated by service. Both Hospital and Tabular views are color-coordinated so that each service is easily located

The Tabular view aids infection analysis by showing epidemiological indicators by place and time (Tasks 1, 2). Users can choose the indicator at different spatial scales: hospital, floor or service level. For by-service, services are color-coded and coordinated with the Hospital view for easy map localization (Fig. 3). Clicking a service pans the Hospital view to that location. With HeatMap activated, this view displays a color legend and weekly aggregated indicator values by service (Fig. 2).

**Fig. 4:**
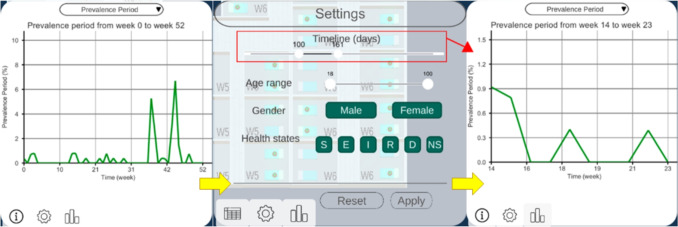
Epi view and filter menu performing semantic zooming. The user can choose a smaller range of time, and this will change the line chart temporal dimension

#### Epi View

The Epi view shows the temporal progression of epidemiological indicators through line charts, allowing analysis of infection evolution (Tasks 1, 4). Users can select an indicator: daily cases and point prevalence, or weekly incidence, period prevalence, mortality rate and incidence density. Daily cases follow the SEIRD-NS model, with health states color-coded to match the Hospital view. This provides an overview of the study period, with options for semantic and graphical zooming (Fig. 4), hovering over charts for specific values and filtering out information (Fig. 1C).

#### Filter of Patients

Besides the view-specific interactions, users can filter patients by age range, gender and health state in all views [26]. They can also zoom, as defined by Shneiderman [26], to focus on a subset of patients, selecting a time range, which automatically updates the other views accordingly (Fig. 4).

### Evaluation Results

In this section, we explain the results of the evaluation regarding usability, interpretability and utility.

#### Participants and Data Collection

The study involved **14 health professionals** (7 women, 7 men) from J. M. Morales Meseguer General University Hospital, Murcia, Spain. Participants ranged in age from 26 to 60, with different years of experience (Table 2). Data collected included responses to CDI scenario tasks, completion times, collaborators’ notes, PSSUQ questionnaire results, and participant feedback.

#### Usability Evaluation

**Fig. 5:**
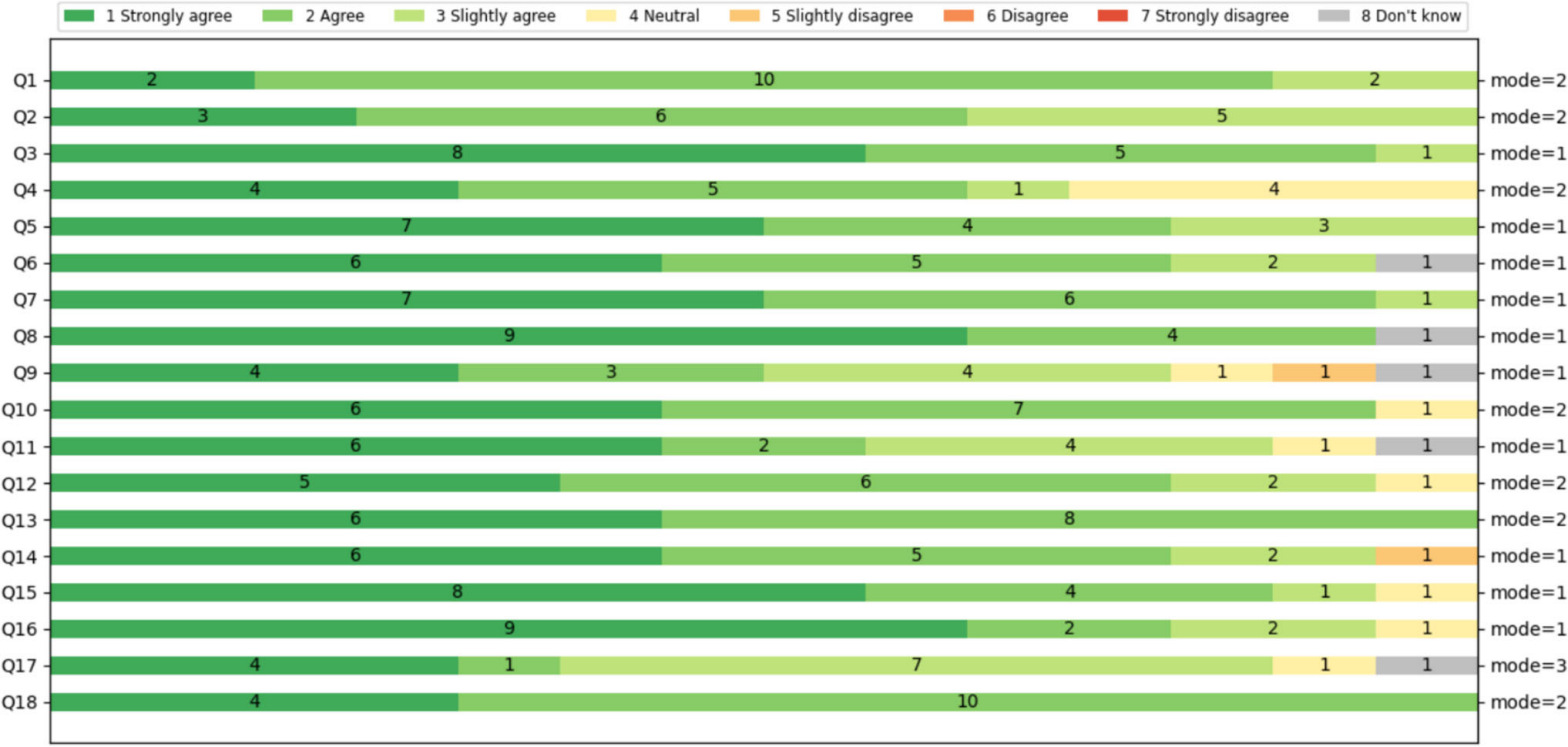
PSSUQ results of OBViz. Q1-Q8 refer to usability, Q9-Q14 refer to quality of information and Q15-Q18 refer to quality of interface

The usability evaluation assessed the tool’s effectiveness, user satisfaction, and ease of use using the PSSUQ. The average response for system usefulness (Q1-Q8) was 1.79 (1 means “totally agree”), indicating high satisfaction (Fig. 5).

**Table 2:** Summary of participants in the evaluation

Age group	25-34	8
	35-44	2
	45-54	2
	$\ge$ 55	2
Gender	Masculine	7
	Femenine	7
Experience (years)	1-5	8
	6-10	2
	$\ge$ 20	4

Participants found the tool easy to use, especially for detecting patient contacts: the ability to see the entire hospital and individual patients to detect contacts was a significant improvement over manual analysis. They also found it intuitive, clear, and quick to learn, with an average response of 1.92 for information quality (Q9-Q14).

Several participants mentioned the interface was pleasant, particularly appreciating the color-coded services for quick location and identification of contaminated beds. For interface quality (Q15-Q18), the average was 1.85. However, some participants noted that patient monitoring was not very intuitive and suggested improvements (see Section 3.2.5).

#### Interpretability Evaluation

Interpretability measures how well the tool explains actions and events in a hospital setting. We evaluated its performance in solving clinical tasks (see Section 2.2) by translating them into 10 daily problems for infection specialists (Appendix C).

**Fig. 6:**
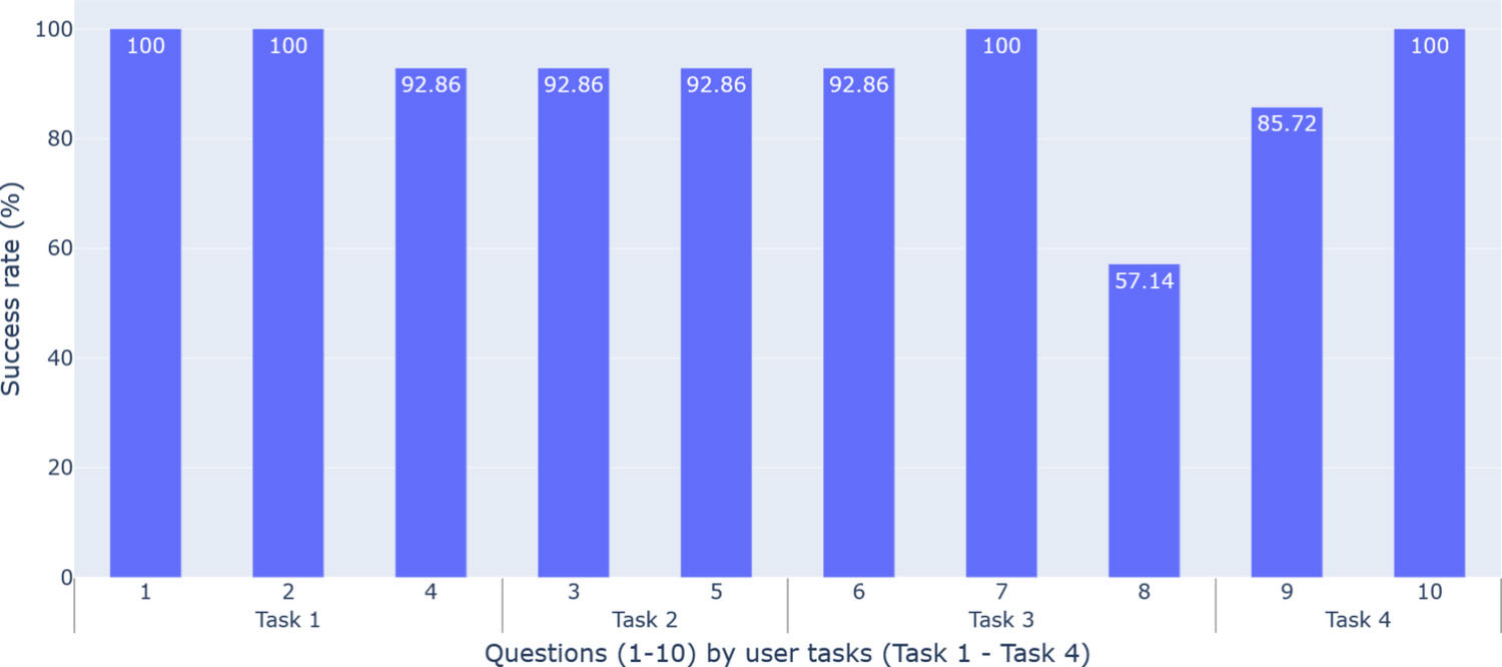
Interpretability results. Success rate of multiple choice questions in the evaluation. Questions (1-10) are categorized by the user tasks to which they belong (Task 1-Task 4)

Figure 6 shows the percentage of correct answers per question, connected to the user task they address. Men had an 88.57% correct rate and women had a 94.29%, with no significant age difference. The lowest score (57.14%) was for patient contact (Q8, Task 3), consistent with participants’ comments on the difficulty of tracking patients.

In question Q9, regarding counting outbreaks (85.72% correct rate), the main challenge was understanding our definition of an outbreak. However, participants who regularly studied hospital outbreaks performed better than those less familiar with the task.

On average, users took 16.36 minutes to complete the questionnaire, with an average success rate of 91.43%.

**Table 3:** Improvements requested

Improvements Requested by users	
*“A patient ID search engine for easier location of specific patients.”*	
*“Patient tracking to trace their movements.”*	
*“Inclusion of healthcare personnel, considered important transmission vectors.”*	
*“Alerts for patient movements or discharges.”*	
*“Patient filtering by infecting microorganism.”*	

#### Utility Evaluation

Utility reflects the interviewee’s perception of the tool’s potential to solve daily tasks.

After testing the tool, we conducted informal interviews to gather opinions, guiding the conversation with pre-established questions when necessary. Ten participants found it useful, and seven indicated they would incorporate it into their workflow. Two saw more utility within the ICU for patient monitoring, while another two expressed interest for annual infection and outbreak studies. Five noted its potential for studying other pathogens (eg., seasonal flu, respiratory viruses).

#### Suggestions for Improvements

During feedback, we asked for suggestions to enhance the tool’s utility. Table 3 shows the most repeated improvements requested.

## Discussion

Many epidemiology tools focus on population-level analyses and geographic visualizations, often for public access. However, there is limited progress in tools designed to help hospital decision-making to control infectious diseases. OBViz combines a 3D hospital visualization, a 2D disease progression, and a tabular visualization for detailed information. This approach uses dynamic and static visualization techniques to show different data perspectives, enabling user interaction, quick analysis and detailed information on demand. The 3D representation can be leveraged with future improvements, such as aggregating information for a spatial axis (eg. aggregate several floors) or showing all the floors from other perspectives.

When developing a visual medical tool, processing real clinical data poses **significant risks**. Using realistic synthetic data, we avoid problems with sensitive and incomplete information. There would be no difference between simulated and real environments if data were obtained with the same precision. The current implementation provides data integration from log files and relational databases, enabling use with both simulated and real data. However, to strengthen the tool’s practical applicability and clinical relevance, further efforts are needed to address the challenges associated with integrating data from electronic health records. These systems often contain heterogeneous, incomplete, and inconsistently structured data, which can hinder tool performance and generalization. Addressing these issues through processes such as data harmonization and robust preprocessing pipelines would enhance the tool’s utility in real-world clinical settings.

Unity was chosen for its efficiency in developing 2D and 3D tools, but has limitations in data visualization, leading to a limited variety of graphics developments in the asset store.

Additionally, **scalability** in hospital modeling is a challenge and possible limitation of this tool. Efforts are underway to automate floor creation. The hospital model presented here was created using this method.

### Validation

is crucial in developing any visualization tool. Not all visual encodings can address every task, so evaluating a visualization’s quality for specific goals is essential. OBViz’s utility, usability and interpretability **were evaluated with healthcare professionals**. The evaluation was carefully planned to optimize professionals’ limited time. In this process, the collaboration with an internist was especially helpful in adapting explanations to the clinicians’ perspective and making us understand it as best as possible.

User evaluation showed that the tool helps specialists perform hospital analysis tasks more efficiently, while patient-level information aids in controlling epidemics within the hospital. Users found the spatial presentation useful and easy to understand, allowing them to quickly locate areas within the hospital. Time-series visualization of disease progression assists in identifying critical periods in a glance. The ability to analyze each hospital service and control time provides flexibility in investigating disease transmission, which is not possible with plain tables or text files, as they currently use. Participants favored the ability to see the hospital overview and then delve into details compared to alternative methods or more abstract visualizations.

The tool successfully resolved 3 out of 4 tasks, except Task 3 (tracking individual patients), reflected in the interpretability results. This is due to the lack of a clearly defined approach for using the tool to find individual patients during the design phase. This has underlined the shortcomings of the tool and improvements, such as highlighting the tracked patient, adding a list of patients who had any contact with them, or indicating when a filter is currently active, so that users do not miss a potentially important group of patients because they are filtered out. Having 14 specialists evaluate the tool has been a great advantage, since they provided perspectives different from ours.

Regarding **utility**, OBViz has multiple applications in health and training. It helps doctors and hospital administrators make informed decisions (eg., isolation of infected patients or greater monitoring of at-risk patients’ movements). It can also be applied in the Objective Structured Clinical Examination, to evaluate medical students’ skills in simulated scenarios. However, utility can be improved by adding different symbols so that people with color vision deficiency can distinguish between patients’ health states.

## Conclusion

We proposed a visual decision support tool that integrates spatial-temporal patient data with their health conditions during an MDR-bacterial infection spread.

The tool combines a 3D hospital visualization, a 2D temporal visualization of the disease, and a tabular visualization for detailed information. Interactivity and animations accurately represent patient movements and infection processes, while the use of known charts facilitates temporal understanding through epidemiological indicators.

The tool was evaluated by 14 health professionals using a PSSUQ questionnaire, with high scores for usability (1.79), information quality (1.92), and interface quality (1.85). These results indicate that the tool was well received by experts. All tasks except Task 3 were successfully completed, demonstrating the clarity and usefulness of the tool in these areas. However, user feedback highlighted areas for improvement, especially in patient tracking and scalability.

## Supplementary Information

Below is the link to the electronic supplementary material.

Supplementary file 1 (mp4 5428 KB)

## Data Availability

The information collected on the evaluation participants and the questionnaires and PSSUQ results are publicly available at http://hdl.handle.net/10201/142060

## Appendix A: Simulation Model and Generated Input Data

The input dataset was generated with H-Outbreak [15], a simulator that allows us to obtain reliable spatial-temporal datasets on hospitalized patients. To achieve this, it combines an agent-based model to represent the patients’ movements and the spread of a CDI in a dynamic population at a low level of abstraction; a macro-model which is a compartmental model to represent the evolution of the infection so it is easy to understand, and the spatial-temporal constraints of a hospital defined by its policies and physical structure.

The evaluation of H-outbreak consisted of an exhaustive process with two phases: verification and validation. During verification, we checked whether the implementation of the conceptual model was correct. During validation, we determined whether the conceptual model was representative of a real-world system with the help of a clinical expert. This process helped us confirm that the infection behaved reasonably, since it resembled CD outbreaks seen in a real hospital. For more information on the simulator design and the evaluation results, we refer the reader to [15].

The generated datasets (presented in Section 2.1) consist of (1) the information of each patient that was admitted to the hospital, and (2) the patients’ trace from their admission to the hospital until their discharge or death from a CDI. The hospital structure is divided into services, which are an Emergency Room (with 20 beds), 3 operating rooms, 5 radiology rooms, an Intensive Care Unit (with 10 beds) and 7 wards. Each ward can contain one or more rooms, and each room has two beds. An example of these datasets can be seen in Tables 4 and 5.

**Table 4:** Patients’ information

ID	Age	Sex	LOS	Admission day
0	59	F	5	1
1	30	F	4	1
2	35	M	7	1

LOS refers to Length of Stay, measured in number of days

**Table 5:** Patients’ movements and infection evolution log

Time step	ID patient	State of health	ID place	Infected place
1	0	Exposed	ER Bed216	False
1	1	Susceptible	Rx room 189	False
1	2	Infected	ER Bed29	True
2	0	Infected	Room90 Bed24	False
2	1	Susceptible	Room8 Bed3	False
2	2	Infected	ER Bed29	True

## Appendix B: Tutorial for OBViz Evaluation

In this section, we present the transcription of the OBViz tool tutorial and the clinical case presented to users who have participated in the evaluation of the tool.

### B.1 Tutorial

The objective of the tool is to help experts in the epidemiological study of a hospital. This includes exploring the spread of infections within hospitals, analyzing the epidemic situation over time, and understanding the information contained in epidemiological indicators. To do this, the tool has 3 linked views. These are:

**3D hospital view:** where you can see the different floors, services and rooms in three dimensions, as well as the spatial location of the patients at all times.

**Tabular view:** where detailed information about each indicator is displayed. This information can be calculated for the entire hospital, by floor or by service.

**Epi view:** where the time trend of each indicator is shown.

What interactions can be performed in each view?

#### Hospital View

In the hospital view, controlled animation of patients can be carried out. With the play/pause button you can start or stop the animation, and the patients will move to wherever they have moved within the hospital at that moment. If we want to advance faster without having to wait for the patients to perform the movement, we can go forward with the +step, +day or +week buttons, then we advance in time and see where they are at each moment automatically. Same with the go backward buttons.

Regarding patients, in addition to movement, we can identify them. The colors in each patient indicate their health state, which takes its values from the SEIRD-NS model. So green is susceptible, yellow is exposed, red is infected, violet is recovered, black is deceased and blue is non-susceptible. Red is also used in beds to indicate that they are contaminated. If we click on a patient, it will show us information about them (age, gender, and length of stay) and if we want to identify them all, we can use the Patient ID option in the toolbar and the ID of each patient will appear on them, and they will follow them if they move.

Regarding the hospital, in the toolbar with the up/down buttons we can change floors and, with the walls/beds/patients checks, we can make these objects appear or disappear in the hospital. We can also perform actions with the camera: with the mouse wheel we can zoom, with the arrows we can move up, down, right or left, and by pressing the right mouse button, we can drag and rotate the view of the hospital.

Another functionality is the heatmap, the indicator chosen will be displayed in the hospital by service, and in the time range chosen in the filtering menu. If the range is very long, it will be divided into weeks. In the tabular view you can see its legend, indicating the maximum and minimum values, and the information in this view will advance by weeks, just like in the hospital.

#### Tabular View

In the tabular view, we will see the information of the indicators change as we move forward in time. We can choose to see the information calculated for the entire hospital or, with the By floor or By service buttons, we will see it aggregated for each floor or for each service. When choosing the per service option, each service in the hospital will be coded with a random color so that it is easily recognizable on the map. If we want to analyze that specific service, we can go directly there by clicking on the item in the tabular view. To exit that view, click the By service button and it will return to the initial version.

#### Epi View

In the epi view, we can choose the indicator between cases, incidence, incidence density, mortality, point prevalence and period prevalence. In this view, the indicator is shown with a line chart for the entire hospital. Here we can also zoom in on an area, if we move the mouse over the lines it will indicate the day or week, and value for that point of time, we can also select in the legend what we do not want to see, and it will disappear from the chart

What interactions are common to all?

The filtering. In the filtered menu you can choose a time range, and filter patients by health status, age or gender, you can apply the filter or reset and return to the original state. This will change all 3 views at once.

### B.2 Clinical case

The clinical case that we are going to work with is the transmission of a Clostridioides difficile infection within the hospital. For this end, the data that we are going to use are generated with a realistic simulator of hospitalized patients. The data represent how patients move through different hospital services and their health changes, according to the SEIRD epidemiological model. In addition to this, there may be non-susceptible patients, that is, patients that are immune to the disease, and therefore will not become infected. The disease can be transmitted from patient to patient or from the environment (bed, room) to the patient and vice versa. Both the evolution of the disease and its transmission follow the epidemiological parameters obtained from scientific literature and experts (example: incubation time, probability of contagion, etc.).

## Appendix C: Questionnaire for the Evaluation of OBViz

In this section, we present the questionnaire used for the evaluation of the OBViz visual tool with healthcare users.

Age:

Gender (M/F):

Years of experience / Years of career:

Specialty:

**Questionnaire**

Choose the right option.

1. In which service was there more mortality in week 1?
  1. Ward0
  2. Ward2
  3. Ward6
2. Which service has the highest incidence of *Clostridioides difficile* during week 19?
  1. Ward0
  2. Ward1
  3. ER
3. What day did the highest concentration (number) of infected cases occur?
  1. Day 340 of the simulation
  2. Day 202 of the simulation
  3. Day 223 of the simulation
4. What was the point prevalence of *Clostridioides difficile *on day 140?
  1. 0%
  2. 5.1%
  3. 3.1%
5. What incidence density did female patients have in week 19 of the simulation? Reset the filter when you finish the question.
  1. 16.8 cases/1000 person-days
  2. 19.9 cases/1000 person-days
  3. 5 cases/1000 person-days
6. The patient with ID 391 gets infected on day 16. What services does he go through while he is infected?
  1. Ward0 and Radiology
  2. ICU and Ward3
  3. ER, Radiology and Ward0
7. What age and gender was the patient with ID 391?
  1. 57 years old and was a man
  2. 31 years old and was a woman
  3. 83 years old and was a woman
8. How many patients was patient ID 391 in contact with (ie, in the same room) while he/she was infected?
  1. 2 patients
  2. 5 patients
  3. 3 patients
9. Defining an infectious outbreak as 3 or more cases that appear in a period of 7 days or less, how many outbreaks were there throughout a year (in the total simulation)?
  1. 5 outbreaks
  2. 10 outbreaks
  3. 14 outbreaks
10. When did the first *Clostridioides difficile* outbreak occur?
  1. On day 5
  2. On day 1
  3. On day 9

## Appendix D: Adapted PSSUQ Questionnaire (1-7)

1 = Totally agree || 7 = Totally disagree

**Table:**

	1	2	3	4	5	6	7
1. Overall, I am satisfied with how easy it is to use this system.							
2. It was simple to use this system. (There weren’t many complications)							
3. I was able to complete tasks effectively using this system.*							
4. I was able to complete tasks quickly using this system.*							
5. I was able to complete tasks efficiently using this system.*							
6. I felt comfortable using this system.							
7. It was easy to learn to use this system.							
8. I believe I could become productive quickly using this system.							
9. The system was clear about what could not be done.							
10. Whenever I made a mistake using the system, I could recover quickly.							
11. The information (such as online help, on-screen messages, and other documentation) provided with this system was clear.							
12. It was easy to find the information I needed.							
13. . The information was effective in helping me complete the tasks and scenarios.							
14. The organization of information on the system screens was clear.							
15. The interface of this system was pleasant.							
16. I liked using the interface of this system.							
17. This system has all the functions and capabilities I expect it to have.							
18. Overall, I am satisfied with this system.							

*Note: **questions 3, 4 and 5** refer to different aspects of completing tasks:

**question 3** refers only to whether the activity can be completed regardless of how long it takes and how complex the task is.

**question 4** asks if it is quick to complete the activity with the tool.

**question 5** (“efficiently”) refers to whether a task that is complex is when few resources are used to complete the task considering that there may be more complex ones.

## Footnotes

1 Available at http://hdl.handle.net/10201/142061

## References

[1] World Health Organization: Antimicrobial resistance, 2023. https://www.who.int/news-room/fact-sheets/detail/antimicrobial-resistance. Accessed 06-March-2024

[2] Serra-Burriel, M., Keys, M., Campillo-Artero, C., Agodi, A., Barchitta, M., Gikas, A., Palos, C., López-Casasnovas, G., Impact of multi-drug resistant bacteria on economic and clinical outcomes of healthcare-associated infections in adults. *PLoS One*. 15(1):0227139, 2020. 10.1371/journal.pone.0227139 10.1371/journal.pone.0227139 PMC6953842 31923281

[3] Jernigan, J.A., Hatfield, K.M., Wolford, H., Nelson, R.E., Olubajo, B., Reddy, S.C., McCarthy, N., Paul, P., McDonald, L.C., Kallen, A., Fiore, A., Craig, M., and Baggs, J., Multidrug-Resistant Bacterial Infections in U.S. Hospitalized Patients, 2012-2017. *New Engl. J. Med.* 382(14):1309–1319, 2020. 10.1056/NEJMoa1914433 10.1056/NEJMoa1914433 PMC10961699 32242356

[4] Cassini, A., Högberg, L.D., Plachouras, D., Quattrocchi, A., Hoxha, A., Simonsen, G.S., Colomb-Cotinat, M., Kretzschmar, M.E., Devleesschauwer, B., Cecchini, M., Ouakrim, D.A., Oliveira, T.C., Struelens, M.J., Suetens, C., and Monnet, D.L., Attributable deaths and disability-adjusted life-years caused by infections with antibiotic-resistant bacteria in the EU and the European Economic Area in 2015. *Lancet Infect. Diseas*. 19(1):56–66, 2019. 10.1016/S1473-3099(18)30605-4 30409683 10.1016/S1473-3099(18)30605-4 PMC6300481

[5] Caban, J.J., Gotz, D., Visual analytics in healthcare – opportunities and research challenges. *J. Am. Med. Inf. Assoc.* 22(2):260–262, 2015. 10.1093/jamia/ocv006 10.1093/jamia/ocv006 PMC11737105 25814539

[6] Rind, A., Miksch, S., Aigner, W., Turic, T., Pohl, M., Visuexplore: Gaining new medical insights from visual exploration. In: *Proceedings of the 1st International Workshop on Interactive Systems in Healthcare (WISH)*, pp. 149–152, 2010

[7] Belay, E.D., Abrams, J., Oster, M.E., Giovanni, J., Pierce, T., Meng, L., Prezzato, E., Balachandran, N., Openshaw, J.J., Rosen, H.E., Kim, M., Richardson, G., Hand, J., Tobin-D’Angelo, M., Wilson, S., Hartley, A., Jones, C., Kolsin, J., Mohamed, H., Colles, Z., Hammett, T., Patel, P., Stierman, B., Campbell, A.P., Godfred-Cato, S., Trends in Geographic and Temporal Distribution of US Children with Multisystem Inflammatory Syndrome during the COVID-19 Pandemic. *JAMA Pediat.* 175(8):837–845, 2021. 10.1001/jamapediatrics.2021.0630 10.1001/jamapediatrics.2021.0630 PMC8025123 33821923

[8] Kissler, S.M., Gog, J.R., Viboud, C., Charu, V., Bjørnstad, O.N., Simonsen, L., Grenfell, B.T., Geographic transmission hubs of the 2009 influenza pandemic in the United States. *Epidemics*. 26:86–94, 2019. 10.1016/j.epidem.2018.10.002 10.1016/j.epidem.2018.10.002 30327253

[9] Oppermann, M., Munzner, T., Ocupado: Visualizing Location-Based Counts Over Time Across Buildings. *Comput. Graph. Forum.* 39(3):127–138, 2020. 10.1111/cgf.13968

[10] Baumgartl, T., Petzold, M., Wunderlich, M., Höhn, M., Archambault, D., Lieser, M., Dalpke, A., Scheithauer, S., Marschollek, M., Eichel, V.M., Mutters, N.T., Consortium, H., Landesberger, T., In Search of Patient Zero. *IEEE Trans. Visual. Comput. Graphics.* 27(2):711–721, 2021. 10.1109/TVCG.2020.3030437 10.1109/TVCG.2020.3030437 33290223

[11] Müller, M., Petzold, M., Wunderlich, M., Baumgartl, T., Höhn, M., Eichel, V., Mutters, N.T., Scheithauer, S., Marschollek, M., Landesberger, T.v., Visual Analysis for Hospital Infection Control using a RNN Model. In: *EuroVA: International Workshop on Visual Analytics*. The Eurographics Association, 2020. 10.2312/eurova.20201090

[12] Sondag, M., Turkay, C., Xu, K., Matthews, L., Mohr, S., Archambault, D., Visual Analytics of Contact Tracing Policy Simulations During an Emergency Response. *Comput. Graph. Forum.* 41(3):29–41, 2022. 10.1111/cgf.14520

[13] Boudreault, L., Hebert-Lavoie, M., Ung, K., Mahmoudhi, C., Vu, Q.P., Jouvet, P., Doyon-Poulin, P., Situation Awareness-Oriented Dashboard in ICUs in Support of Resource Management in Time of Pandemics. *IEEE J. Transl. Eng. Health Med.* 11:151–160, 2023. 10.1109/JTEHM.2023.3241215 10.1109/JTEHM.2023.3241215 PMC9904450 36816098

[14] World Health Organization: Ethics and governance of artificial intelligence for health, 2021. https://www.who.int/publications-detail-redirect/9789240029200 Accessed 08-March-2023

[15] Kim, D., Canovas-Segura, B., Jimeno-Almazán, A., Campos, M., Juarez, J.M., Spatial-temporal simulation for hospital infection spread and outbreaks of Clostridioides difficile. *Sci. Rep.* 13(1), 2023. 10.1038/s41598-023-47296-1 10.1038/s41598-023-47296-1 PMC10654661 37974000

[16] Hota, S.S., Achonu, C., Crowcroft, N.S., Harvey, B.J., Lauwers, A., Gardam, M.A., Determining Mortality Rates Attributable to Clostridium difficile Infection. *Emerg. Infect. Diseas.* 18(2):305–307, 2012. 10.3201/eid1802.101611 10.3201/eid1802.101611 PMC3310441 22305427

[17] Lital Meyer, S., Ricardo Espinoza, A., Rodrigo Quera, P., Infeccion por clostridium difficile. *Revista Medica Clinica Las Condes. *25(3):473–484, 2014. 10.1016/S0716-8640(14)70064-1

[18] Brauer, F., Compartmental Models in Epidemiology. *Math. Epidemiol.* 1945:19–79, 2008. 10.1007/978-3-540-78911-6_2

[19] Centers for Disease Control and Prevention: General Recommendations for Routine Prevention and Control of MDROs in Healthcare Settings, 2019. https://www.cdc.gov/infectioncontrol/guidelines/mdro/table3-1-routine-prevention.html Accessed 06-March-2024

[20] Munzner, T., A Nested Model for Visualization Design and Validation. *IEEE Trans. Visual. Comput. Graph.* 15(6):921–928, 2009. 10.1109/TVCG.2009.111 10.1109/TVCG.2009.111 19834155

[21] Ice Pond Studio. https://www.icepondstudio.com/ Accessed 29-Feb-2024

[22] Lewis, J., Psychometric Evaluation of the PSSUQ Using Data from Five Years of Usability Studies. *Int. J. Human-Comput. Inter.* 14:463–488, 2002. 10.1080/10447318.2002.9669130

[23] Wu, D.T.Y., Chen, A.T., Manning, J.D., Levy-Fix, G., Backonja, U., Borland, D., Caban, J.J., Dowding, D.W., Hochheiser, H., Kagan, V., Kandaswamy, S., Kumar, M., Nunez, A., Pan, E., Gotz, D., Evaluating visual analytics for health informatics applications. *J. Am. Med. Inf. Assoc. JAMIA*. 26(4):314–323, 2019. 10.1093/jamia/ocy190 10.1093/jamia/ocy190 PMC7647177 30840080

[24] Aigner, W., Miksch, S., Schumann, H., Tominski, C., *Visualization of Time-Oriented Data. Human-Computer Interaction Series*. Springer, London, 2011.

[25] Munzner, T., *Visualization Analysis and Design*. CRC Press, USA, 2014

[26] Shneiderman, B., The eyes have it: a task by data type taxonomy for information visualizations. In: *Proceedings 1996 IEEE Symposium on Visual Languages*, pp. 336–343, 1996. 10.1109/VL.1996.545307

